# Assessing the functions underlying learning using by-trial and by-participant models: Evidence from two visual perceptual learning paradigms

**DOI:** 10.1167/jov.21.13.5

**Published:** 2021-12-14

**Authors:** Aaron Cochrane, C. Shawn Green

**Affiliations:** 1 Faculty of Psychology and Education Sciences, University of Geneva, Geneva, Switzerland; 2 Department of Psychology, University of Wisconsin–Madison, Madison, WI, USA

**Keywords:** perceptual learning, learning function, continuous-time models

## Abstract

Inferred mechanisms of learning, such as those involved in improvements resulting from perceptual training, are reliant on (and reflect) the functional forms that models of learning take. However, previous investigations of the functional forms of perceptual learning have been limited in ways that are incompatible with the known mechanisms of learning. For instance, previous work has overwhelmingly aggregated learning data across learning participants, learning trials, or both. Here we approach the study of the functional form of perceptual learning on the by-person and by-trial levels at which the mechanisms of learning are expected to act. Each participant completed one of two visual perceptual learning tasks over the course of two days, with the first 75% of task performance using a single reference stimulus (i.e., “training”) and the last 25% using an orthogonal reference stimulus (to test generalization). Five learning functions, coming from either the exponential or the power family, were fit to each participant's data. The exponential family was uniformly supported by Bayesian Information Criteria (BIC) model comparisons. The simplest exponential function was the best fit to learning on a texture oddball detection task, while a Weibull (augmented exponential) function tended to be the best fit to learning on a dot-motion discrimination task. The support for the exponential family corroborated previous by-person investigations of the functional form of learning, while the novel evidence supporting the Weibull learning model has implications for both the analysis and the mechanistic bases of the learning.

## Introduction

Given proper experience, humans tend to show significant improvements in the ability to make many different types of perceptual decisions (e.g., discrimination, detection categorization, estimation) about a wide variety of stimuli (e.g., from basic black and white gratings or moving dots to radiological images or faces). Of particular interest to researchers, beyond the simple fact that improvements are observed, is the functional relationship between the amount of experience and the degree of improvement. Indeed, understanding the mathematical functions that best capture learning is not simply an empirical data-fitting problem with the goal of providing purely descriptive outcomes. Instead, uncovering the best-fitting functional form for learning has deep inferential repercussions. For instance, certain mechanistic accounts of learning produce very characteristic shapes (e.g., the influential ACT-R system produces a power function; Anderson et al., 1999). As such, identifying the functional form of learning shown by human observers on different types of tasks provides leverage when considering the most likely underlying mechanisms.

**Figure 1.**
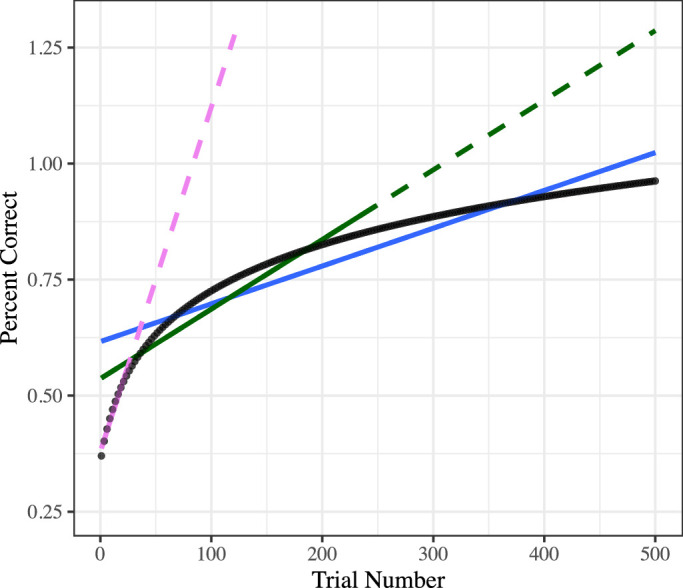
Implications of the timescale-dependent biases of linear fits. While a linear relationship is clearly biologically implausible in the limit, even in smaller time windows, an inappropriate assumption of linearity can create problems. In the example above, a given “true” learning curve (black dotted line) is plotted alongside solid lines of linear fits to 50 trials (purple), 250 trials (green), or 500 trials (blue). Critically, despite each being fit to the same actual learning process, the various linear models provide very different inferences regarding the nature of learning. The 50-trial linear fit necessarily indicates rapid learning that, if extrapolated from (dotted line continuation), quickly reaches impossibly large values of accuracy. The 500-trial linear model meanwhile necessarily fits a flatter line (i.e., putatively “slower” change) than the models fit to a smaller number of trials. The 500-trial model thereby misses, by design, the early rapid changes of the learning curve. The slopes, beginning levels, and ending levels of performance diverge between all models. All models extrapolate to impossible levels of accuracy (i.e., over 100%).

Interestingly, in large swaths of literature, learning is frequently (at least implicitly) modeled as being linear with time (e.g., via the use of change scores such as pretest mean minus posttest mean, linear trends such as using trial or session number as a covariate, certain growth curve models, etc.). While in many cases, such analyses are likely employed based on ease or convenience, if learning is not in fact linear with time, it can be a threat to the validity of the analyses (e.g., outcomes can depend in a problematic fashion on the number of trials that are considered; see Figure 1).

Given the obvious issues with linear accounts, when researchers have attempted to directly characterize the functional form of learning, candidate functions have nearly always been nonlinear and saturating (i.e., the function reaches some true asymptote or maximum in performance; Newell et al., 2009). Of the possible functional forms, historically the most dominant has been the power function (e.g., Anderson et al., 1999; Crossman, 1959; Newell & Rosenbloom, 1981; Snoddy, 1926; Stratton et al., 2007). In fact, the ubiquity with which power functions were found to appropriately describe learning data led to the moniker “power law of learning.” Yet more recently, the appropriateness of power functions as an empirical description of learning has been called into question (Heathcote et al., 2000; Stratton et al., 2007). One of the primary arguments therein has been that while power functions often provide a good fit to group-level aggregates (i.e., when averaging across many participants' learning curves), other functional forms are more appropriate for describing the learning curves of individual participants. This was seen, for instance, in seminal work by Dosher and Lu (2007) in the perceptual domain, who found that exponential functions provided a superior fit to individual perceptual learning data as compared to power functions (see also Heathcote et al., 2000).

Our aim here was to extend the consideration of the functional form of perceptual learning in three distinct ways from the previous literature. First, while previous work has argued that exponential functions are the most appropriate for fitting individual perceptual learning data, most of these assessments have nonetheless used some degree of aggregation across learners and/or across trials (e.g., Dosher & Lu, 2007), leaving uncertainty about the best-fitting shape at the individual trial-by-trial level (e.g., the best-fitting shape to the five points given by the average of trials 1–100, 101–200, 201–300, 301–400, and 401–500 will not necessarily be the best-fitting shape when considering trials 1–500 continuously). Previous work by our group has demonstrated the theoretical and empirical justifications for implementing continuous-time models of learning (i.e., without aggregating data at the level of trials or individuals; Kattner, Cochrane, Cox, et al., 2017; Kattner, Cochrane, & Green, 2017; for similar arguments regarding trial-by-trial variation, see Kumar & Glaser, 1993; Zhang et al., 2019a). Indeed, the arguments for disaggregated analyses of learning can be made succinctly a priori: Because theories of learning consider learning that accumulates with each learning event (i.e., trial) and within individual learners, inferences regarding such learning should consider by-trial and by-individual learning trajectories.

Instead, the use of sparse “block-level” performance (e.g., psychometric function or staircase point estimates) implies within-block stationarity of performance while overfitting trajectories of learning. Overfitting arises from the disregard for degrees of freedom in generating point estimates, when using stepwise procedures, that imply noise-free estimates of performance (Kattner, Cochrane, & Green, 2017). Implicit within-block stationarity precludes certain questions regarding the functional form of learning. Variations in learning curves occurring in short periods of time (i.e., intrablock) cannot be captured; this is especially likely to be the case early in learning. Yet, while in our previous work, we have shown that these time-continuous methods (implementing an exponential form) outperformed aggregation-based fitting techniques, we did not have the data in this previous work that were necessarily to adjudicate between other possible forms (e.g., power functions). As such, our first goal here was to examine whether, using time-continuous fitting methods, there was more support for exponential than power functional forms of perceptual learning (which would provide converging support for the arguments made by Dosher & Lu, 2007, among others).

Here we note that our specific approach differs from, while also complementing, other research that has attempted to examine perceptual learning in a more continuous fashion. This includes work by Zhang et al. (2019b), in which stimuli during learning were controlled by an adaptive algorithm. While there are strong virtues to such systems (e.g., in terms of efficiency and model-based task design), they nonetheless create confounds between participant response, trial number, and stimulus strength (i.e., in making stimulus strength the outcome measure of interest). These confounds have the potential to then influence the process of learning itself (Ahissar & Hochstein, 1997; Hung & Seitz, 2014; Lin et al., 2017; Wang et al., 2013) and, as such, could also influence the functional form. The adaptive stimulus approach stands in contrast with the methods employed here, which used a constant range of stimuli over the duration of learning and in which participant behavior is the outcome measure (although this latter approach presents its own confound with difficulty, namely, that the task becomes easier for participants as they learn).

Second, previous work has frequently considered just the “base” (i.e., three-parameter) form of the exponential and power families. These produce very characteristic shapes that would constrain the possible mechanistic underpinnings. For instance, exponential decay, in which the proportion of change remaining is a function of time elapsed (i.e., constant hazard rate), implies a single mechanism of change (i.e., each unit of input [time] produces one particular proportional amount of change); power-law functions, in contrast, imply multiple averaged or cascading mechanisms of change (as the rate of change slows per unit [time]). Yet, there are augmentations to the base parameterizations that allow for the examination of specific additional hypotheses (i.e., these do not just add free parameters and thus additional flexibility in fitting; the additional parameters also carry clear and interpretable meanings). In the case of power functions, one common example is an additional parameter to model theoretical “quantity of prior learning” (Heathcote et al., 2000). Additions to the exponential function allow, for example, modeling of multiple learning processes using several rate parameters (Newell et al., 2009; Reddy et al., 2018) or an initial acceleration or “slow start” of learning (Brooks et al., 1995; Leibowitz et al., 2010; Newell et al., 2001). The cumulative Weibull function adds a single shape parameter to the three-parameter exponential function and thereby allows for an interpolation between the simplest exponential function and a fully sigmoid function akin to a logit or probit (Gallistel et al., 2004; Leibowitz et al., 2010). The shape parameter corresponds to a deceleration or acceleration of the hazard, with a more sigmoid-like function indicating a slow start to learning and an accelerating hazard rate (potentially corresponding to various empirical suggestions of “early” and “late” stages of perceptual learning; e.g., Fahle, 2005; Shibata et al., 2014). As such, our second goal was to examine whether any such augmented versions of the functional forms provided better fits than the three-parameter versions (when appropriately penalized for the additional flexibility).

Third, and last, previous work on the functional forms of perceptual learning has generally focused exclusively on learning on the initial trained task. However, the extent to which perceptual training transfers to new stimuli/tasks is another major consideration in the domain of perceptual learning. Typically, though, learning generalization has been tested via a single block of a new task, with performance on the new task being aggregated over the entire block (e.g., trained for 1,000 trials on Task 1 and then generalization assessed via 100 trials on Task 2). In previous work, we have shown that this type of procedure conflates two independent ways in which learning can potentially generalize—“immediate transfer” (where training on Task A produces an immediate benefit on Task B) and “learning to learn” (where training on Task A does not produce an immediate benefit to Task B but does allow Task B to be learned more quickly than Task A). Because these two routes to learning generalization may be associated with very different mechanisms, it is important to differentiate between them. As such, our third goal was to examine the functional form of learning generalization, in a time-dependent fashion, in conjunction with an investigation of the functions characterizing initial learning.

In this current work, we took inspiration from the perceptual learning studies of Ahissar and Hochstein (2000) and Wang and colleagues (2013), who each demonstrated differential learning generalization as a function of task difficulty. Specifically, greater learning generalization has been seen when participants were trained on easier as compared to harder versions of the tasks (Ahissar & Hochstein, 1997; Liu, 1999; although see also Jeter et al., 2009; Cohen & Weinshall, 2017, whose empirical-data/computational modeling has suggested the true driving force of the phenomenon is the difficulty of the generalization tasks rather than the training tasks). The enhanced generalization in response to easier training regimes was then in turn seen as evidence for distinct mechanistic loci of learning, with mechanisms of learning on easy tasks being more general and mechanisms of learning on difficult tasks being more specific (reviewed in Ahissar et al., 2009). However, previous work examining difficulty-influenced generalization relied on aggregated measures of performance, thereby possibly conflating processes of generalization that may have been time dependent (i.e., initial performance vs. learning to tune visual perception to novel stimuli; see Ahissar et al., 2009, Figure 4). Although some time-evolving aspects of difficulty and generalization have been considered, such as linear models fit to block-level d-prime (Wang et al., 2013), no previous examination of generalization has used functionally appropriate methods sensitive to rapid changes in performance. By using by-trial models of the most empirically appropriate functions, we specifically tested for two possible routes to difficulty modulations in generalization. First, we tested whether immediate benefits of previous training would be evident at the start of generalization. Second, we tested whether learning within the generalization task would be faster or slower as a function of training task.

## Methods

### Participants and materials

We recruited participants (*n* = 132, *M*_age_ = 19.0, *SD*_age_ = 0.7, 70 female, 72% White, 13% Asian, 15% other, multiple, or no response) from the University of Wisconsin–Madison Introduction to Psychology participant pool. All participants read and signed consent forms and were compensated with course credit. All procedures comply with the Declaration of Helsinki and were approved by the University of Wisconsin–Madison Institutional Review Board.

**Figure 2.**
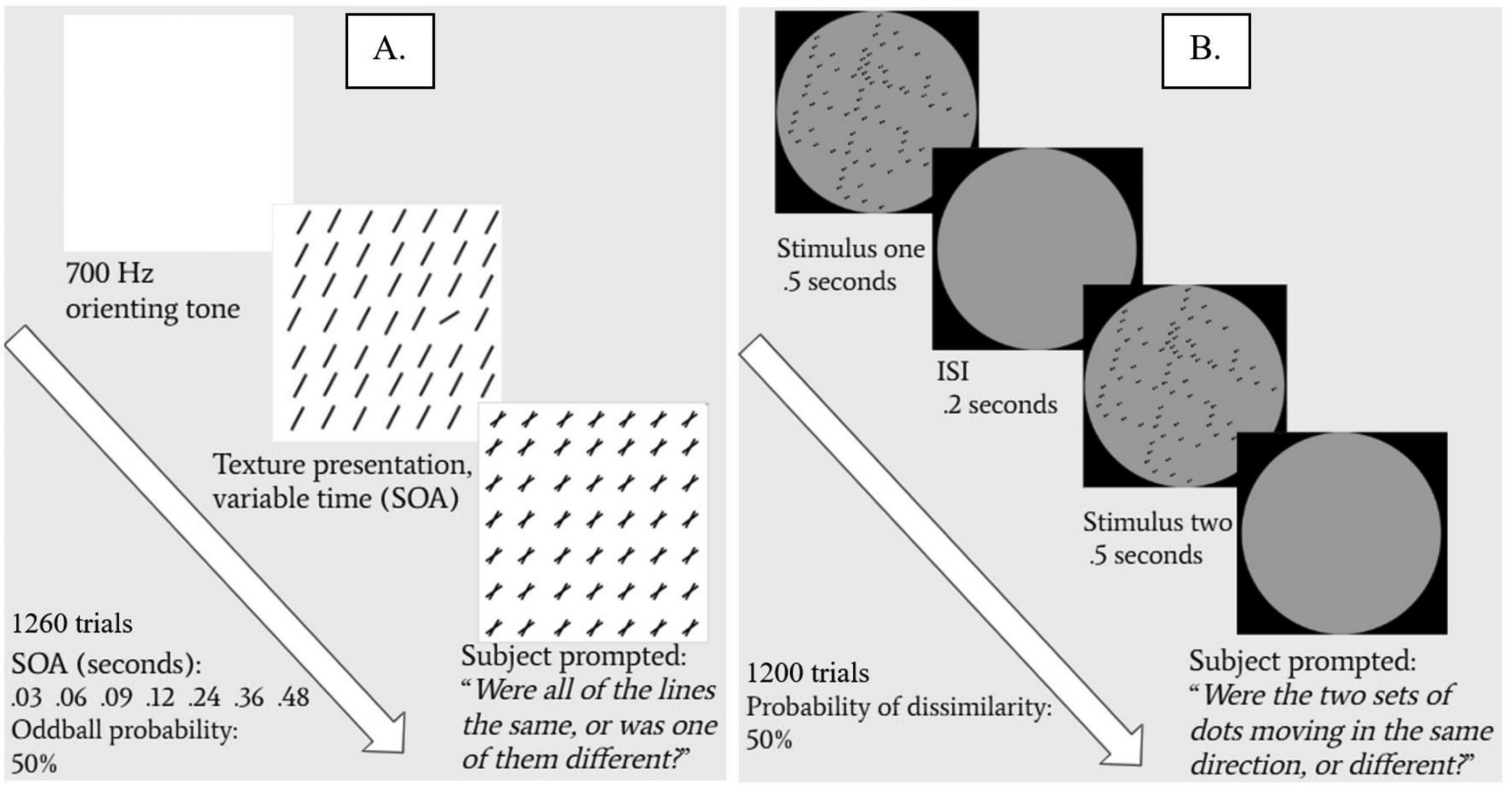
Depictions of each perceptual learning paradigm. (A) Texture oddball detection task and (B) dot-motion direction discrimination task. For further details, see the Supplementary Material. ISI = XXX.

Participants were assigned to one of four groups. Two groups completed a texture oddball detection task (Ahissar & Hochstein, 1993, 2000; see Figure 2A) while two groups completed a dot-motion direction discrimination task (Ball & Sekuler, 1987; Liu, 1999; Wang et al., 2013; see Figure 2B & Supplementary Material). Within each of these tasks, one group completed an easier version (26 texture oddball detection, 31 dot-motion direction discrimination) while one group completed a more difficult version (30 texture oddball detection, 45 dot-motion direction discrimination). Participants were assigned pseudorandomly to one difficulty group. The recruitment target was 20 retained participants per condition; after exclusions, we closely matched this target (see Results).

All stimuli were presented on a 22-in. Dell monitor using Psychtoolbox 3 (Brainard, 1997; Kleiner et al., 2007) in MATLAB, on a Dell PC running Windows 10. Viewing distance was approximately 59 cm and was not fixed.

### Procedure

All participants completed two days of training involving eight blocks of a perceptual learning task. Short breaks were allowed between each block. The first six blocks (four on the first day and two on the second) involved training on a single stimulus reference orientation (i.e., texture orientation or mean dot-motion direction). The last two blocks tested generalization of learning by training on an orthogonal reference orientation. Before the first block and the seventh block, each participant completed four trials with very large orientation offsets and slower timing in order to familiarize them with the reference orientations within the phase of the task. Training stimulus sets remained constant throughout the experiment.

Two perceptual learning tasks, modeled after Ahissar and Hochstein (2000) and Wang, Zhou, and Liu (2013; only within-difficulty generalization), were employed in separate groups of participants. Task parameters were largely taken from these studies (see also Figure 2). Two difficulty orientation offsets were used in each task: easy (texture: 30°; motion: 8°) and difficult (texture: 16°; motion: 4°). All participants were trained for approximately one hour on each of two days (840 per day texture, 800 per day motion). The first 75% of trials used one reference angle (16° texture, 40° motion), while the last 25% of trials tested for generalization to another reference angle (106° texture, 130° motion). Additional task information is reported in the Supplementary Material.

### Analysis

Nonlinear learning models were fit in R using the **TEfits** package (Cochrane, 2020 model code reported in the Supplementary Material). As in the original studies cited above, outcomes in texture detection were defined as thresholds while outcomes in dot-motion were defined as d-prime. Learning functions are described in detail in the **TEfits** documentation. Note that in Ahissar and Hochstein (2000), texture detection thresholds were fit using the “Quick function,” an alternative parameterization of the Weibull function (Strasburger, 2001), whereas **TEfits** uses a numerically different but functionally equivalent parameterization of the same psychometric function (see **TEfits** documentation). **TEfits** threshold values are parameterized as the stimulus strength (i.e., Stimulus Onset Asynchrony (SOA)) necessary to achieve 75% accuracy. For the dot-motion direction discrimination data, we first used the **TEfits** function **tef_acc2dprime** to calculate a by-trial d-prime using a Gaussian kernel–weighted percent hits and false alarms (kernel half-width half-max of two trials). Vectors of d-primes were estimated for each participant's training and generalization separately, and then the d-primes from this vector were fit using a least squares (i.e., maximum likelihood) loss function. We note that, while we did induce some smoothing and loss of temporal precision by using this Gaussian kernel, in practice, a two-trial half-width half-max induces very little smoothing and is much smaller than any typical blockwise analysis of learning. Texture oddball detection data were fit by maximum likelihood as well (i.e., minimizing the error of model predictions given the Bernoulli likelihood function). Maximum likelihood estimation utilized 2,000 randomly initialized parameter combinations, each followed by a Broyden–Fletcher–Goldfarb–Shanno (BFGS) optimization run, for each participant for each model, to increase the chances that the best-fitting model was indeed the global likelihood maximum rather than a local maximum.

Analyses were guided by the goal of identifying the best-fitting mathematical function describing changes in performance associated with practice. The primary candidate functional forms of learning included three-parameter power and exponential functions, as examined in similar previous studies (Crossman, 1959; Dosher & Lu, 2007; Heathcote et al., 2000; Leibowitz et al., 2010; Newell & Rosenbloom, 1981; Newell et al., 2009; Snoddy, 1926). Broadly, the exponential family takes the form in Equation 1. In contrast, the power family takes the form in Equation 2.

(1) $$start+{\left(asymptote-start\right)}^{time\times rate}\;$$

(2) $$start+\left(asymptote-start\right)\;\times time^{rate}$$

Each of these functions allows a parameterization with three free parameters describing (a) the starting point of performance (i.e., y-intercept at Trial 1), (b) the rate or time constant of change, and (c) the asymptotic level of performance expected with an infinite amount of experience. A common augmentation of the power function includes an extra parameter, conceptualized as “amount of previous experience” (Heathcote et al., 2000), which adds a great deal of flexibility to the shape of the power function (see Equation 3; note that the final multiplicative term is a normalizing constant to allow clear interpretation of starting and asymptotic values).

(3) $$\;\;\;\;\;\;\begin{array}{c} start+\left(asymptote-start\right)\times \;{\left(time+previousTime\right)}^{rate}\;\times \frac{1}{previousTime^{rate}} \end{array}$$

Two four-parameter extensions of the exponential function were also tested: (a) an equally weighted combination of two exponential functions (Reddy et al., 2018; see Equation 4) and (b) a Weibull function (Gallistel et al., 2004; see Equation 5). The Weibull function is of particular interest; it is an extension of the exponential function to learning that may start quickly and decelerate or start slowly, accelerate, and form a sigmoid learning function.

(4) $$\;\;\;\;\;\;\;\;\;\;\;\;\begin{array}{c} start\;+\;.5\;\times \;{\left(asymptote-start\right)}^{time\times rate_{1}}\;+\;.5\;\times \;{\left(asymptote-start\right)}^{time\times rate_{2}} \end{array}$$

(5) $$start+{\left(asymptote-start\right)}^{{\left(time\times rate\right)}^{shape}}\;$$

Each of the equations above shows the simplest representation of each functional form; in practice, each parameter of the functions in Equations 1 to 5 was monotonically transformed (e.g., sign-flipped or log-transformed) for ease of estimation and interpretation (see Supplementary Material). These transformations should have no impact on goodness-of-fit indices. All functional forms included starting and asymptote parameters, with differences between families and functions occurring in the shape of the trajectory of learning between start and asymptote. Within **TEfits**, using maximum likelihood estimation, all parameters are constrained to be within reasonable values (i.e., the likelihood function overwhelmingly penalizes unreasonable values). Most important, the start and asymptote parameters must be within a liberal range of possibility; in this case, thresholds were limited to be between 0 and .98 in the texture task (i.e., between zero and two times the maximum SOA), and d-prime was constrained between 0 and 5 in the dot-motion task (corresponding to percent correct ranging from chance, 50%, to approximately 99.38%, or perfect accuracy with a .62% lapse rate; see Supplementary Material for model code). Restrictions on other parameters (e.g., rate) should be less influential on the outcome of the fitting, with the primary precluded outcome being that “learning” could not happen in an extremely small number of trials (e.g., half of learning in two trials). This restriction on rate parameters assists in the estimation of nonlinear functions by reducing the ability for combinations of parameters to imitate one another (i.e., if performance changes very little, this should not be fit as reaching asymptote in two trials, with a potentially large difference between start and asymptote; instead, it should take the form of starting performance being nearly identical to asymptotic performance).

**Figure 3.**
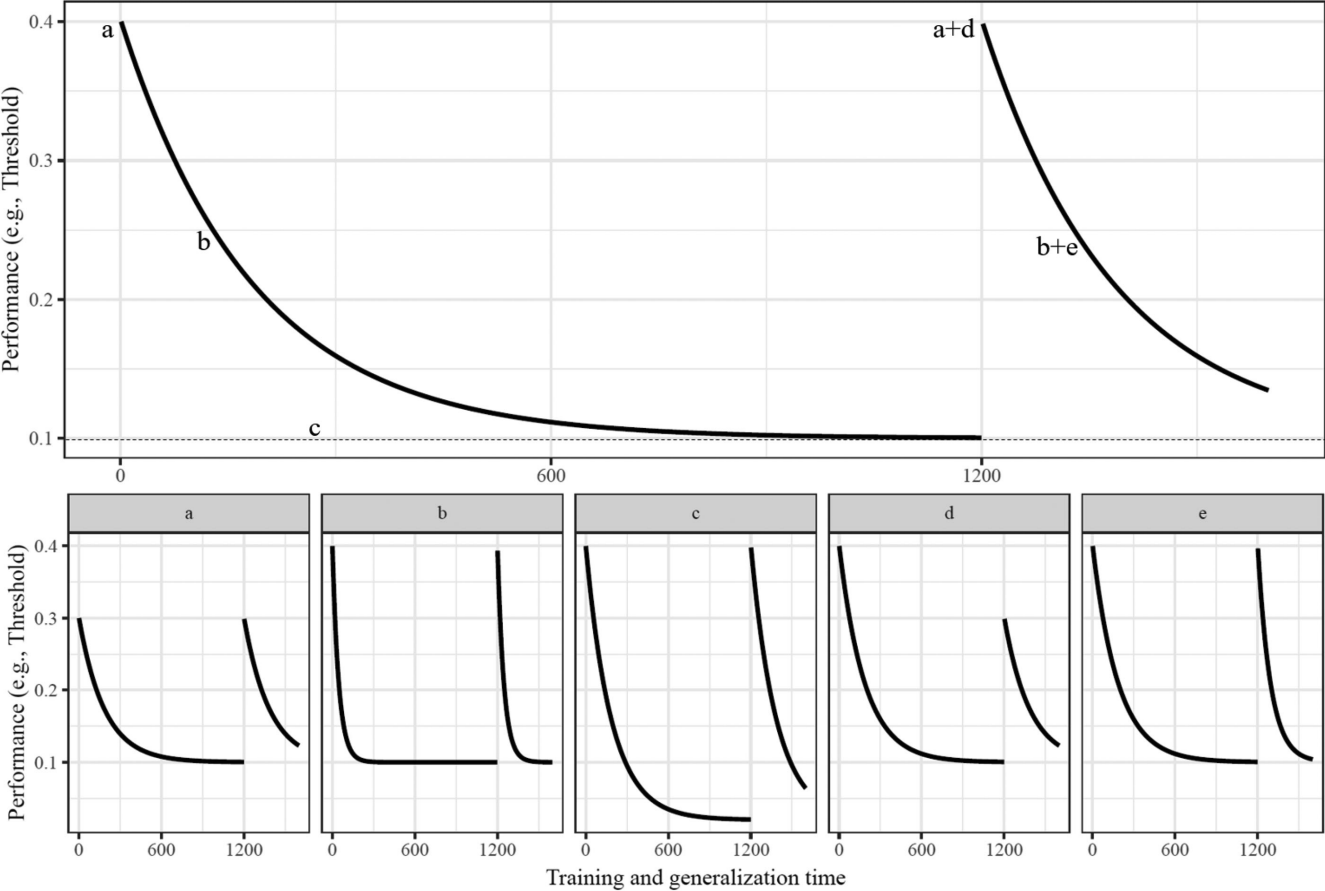
Example parameterization of a three-parameter exponential model (with the three-parameter power model using parameters with the same implications). Black line represents estimated performance on a given trial (with the parameter values of a = .4, b = 7.0, c = .1, d = 0, and e = 0). All models included these parameters, with more complex models having additional flexibility in shape [rate]. Each bottom panel changes the single labeled parameter (all other parameters identical to the top panel). Variations in parameters included (a) starting point of d-prime or threshold [0.3]; (b) rate of change in initial learning [5], specified as a log time constant; (c) asymptotic d-prime or threshold [0.02]; (d) difference between d-prime or threshold initial training start and generalization start [−0.1]; and (e) difference between rate of change in initial training and rate of change in generalization [−1].

Assessment of functional forms necessitated fitting parameters at the levels of individual participants (Dosher & Lu, 2007; Heathcote et al., 2000; Stratton et al., 2007) and using time as a continuously varying dimension (Kattner, Cochrane, & Green, 2017). Within participants, each possible model of interest was fit simultaneously to both initial training and test of generalization (see Figure 3 for examples). This included common parameters between training and generalization *except* for initial ability and rate of learning. Start and rate parameters were estimated as varying between training and generalization, thereby allowing for tests of generalization. Note that, in the Results, we also report comparisons of fits to only the initial training. Representative learning curves for each function can be observed in the Results. After obtaining a Bayesian Information Criterion (BIC) value for each model for each participant (optimized using **TEfits**), model comparisons were conducted by first normalizing model BIC within participants to extract Schwarz weights (Wagenmakers & Farrell, 2004). Schwarz weights must sum to 1 within participants, with the highest weight indicating the best-fitting model.

Secondary statistical analyses, following the primary Schwarz weight comparisons, primarily used bootstrapped robust linear models (i.e., **tef_rlm_boot** from the **TEfits** package). These models fit robust linear models, using the **R** package **MASS** (Venables & Ripley, 2002) to each of 2,000 data sets resampled with replacement to inform the 95% CI. Furthermore, the models fit robust linear models to each of 2,000 subsets of data (random 80% without replacement) and used the fit model to predict the held-out 20% of data. The median proportional reduction in least squares error in the held-out data was reported as Δ*R*^2^_oos_.

## Results

### Results: Data processing and exclusions

**Figure 4.**
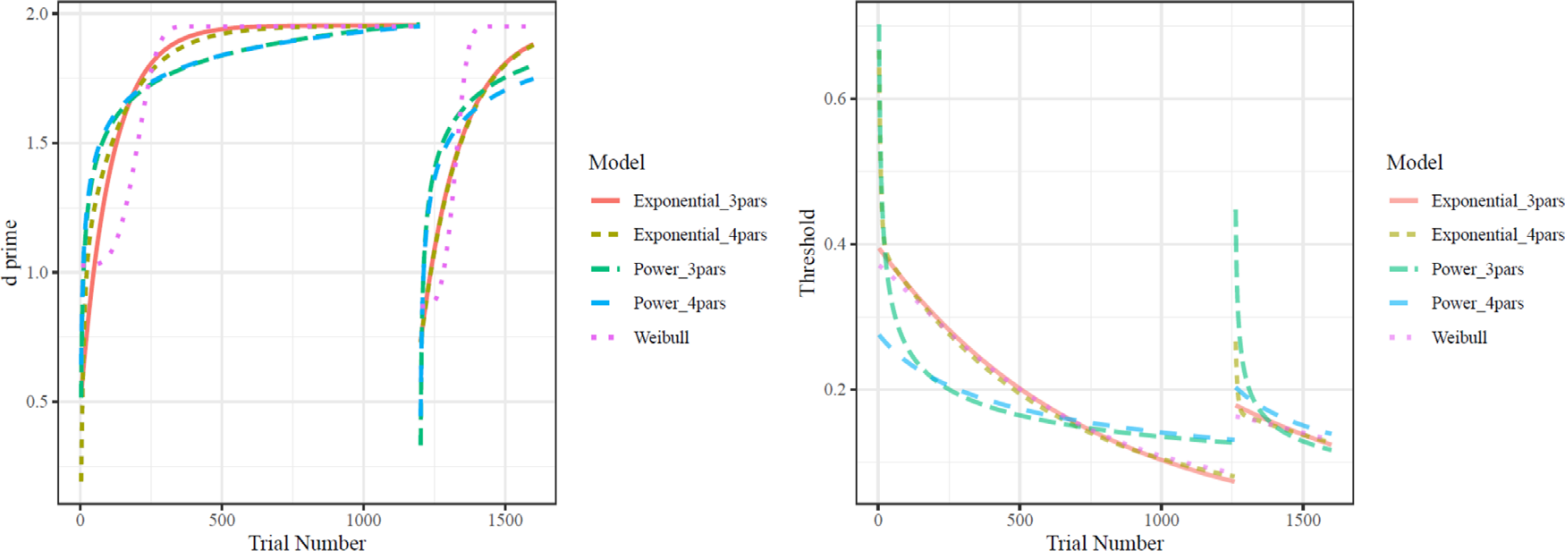
Example predicted time-evolving d-prime (left panel) and psychometric function threshold (right panel) from each learning function fit to one participant for each task. The x-axis is overall trial number, including both initial training and generalization (the disjunction in the curves occurs on the trial in which participants switch from initial training to the generalization task). In the left panel, the y-axis is d-prime, or sensitivity, with greater d-prime indicating superior performance. In the right panel, the y-axis is a Weibull psychometric function threshold indicating the number of seconds needed to achieve 75% accuracy. Lower threshold values indicate better performance. Learning curves display characteristic differences between the power family (Power_3pars and Power_4pars) and the exponential family (Exponential_3pars, Exponential_4pars, and Weibull). In the left panel, the Weibull function is strikingly different from the other four functions, however, by allowing for an initial acceleration of learning and a sigmoid shape. Note that overlapping predictions, arising from models from the same family, may be difficult to discern.

The texture oddball detection task started with 26 easy-condition participants and 30 hard-condition participants. Six easy-condition participants and 11 hard-condition participants were excluded due to failures to learn. Specifically, this included participants who did not achieve above-chance performance on their final 200 training trials (i.e., failing to reject the null of a one-tailed binomial test with a guessing rate of 50%) or had accuracy on the final 200 trials of training that was lower than accuracy on the first 200 trials of training. Each of these exclusion criteria was calculated on raw percent correct data prior to fitting any models. This left 20 easy-condition participants and 19 hard-condition participants. The dot-motion perceptual learning started with 31 easy-condition participants and 45 hard-condition participants. Eight easy-condition participants and 25 hard-condition participants were excluded due to (a) not achieving above-chance performance on their final 200 training trials or (b) having accuracy on the final 200 trials of training that was lower than accuracy on the first 200 trials of training. This left 23 easy-condition participants and 20 hard-condition participants. The rate of exclusions was quite high, particularly in the dot-motion perceptual learning. The high number of exclusions was unfortunate, but the current study of learning necessitated a confirmation that all participants learned (i.e., parameterizations of the learning function are only sensible if participants learned). Figure 4 shows the fit values of each learning function to data from an example participant from each study.

### Results: Best-fitting models to learning and generalization data

**Figure 5.**
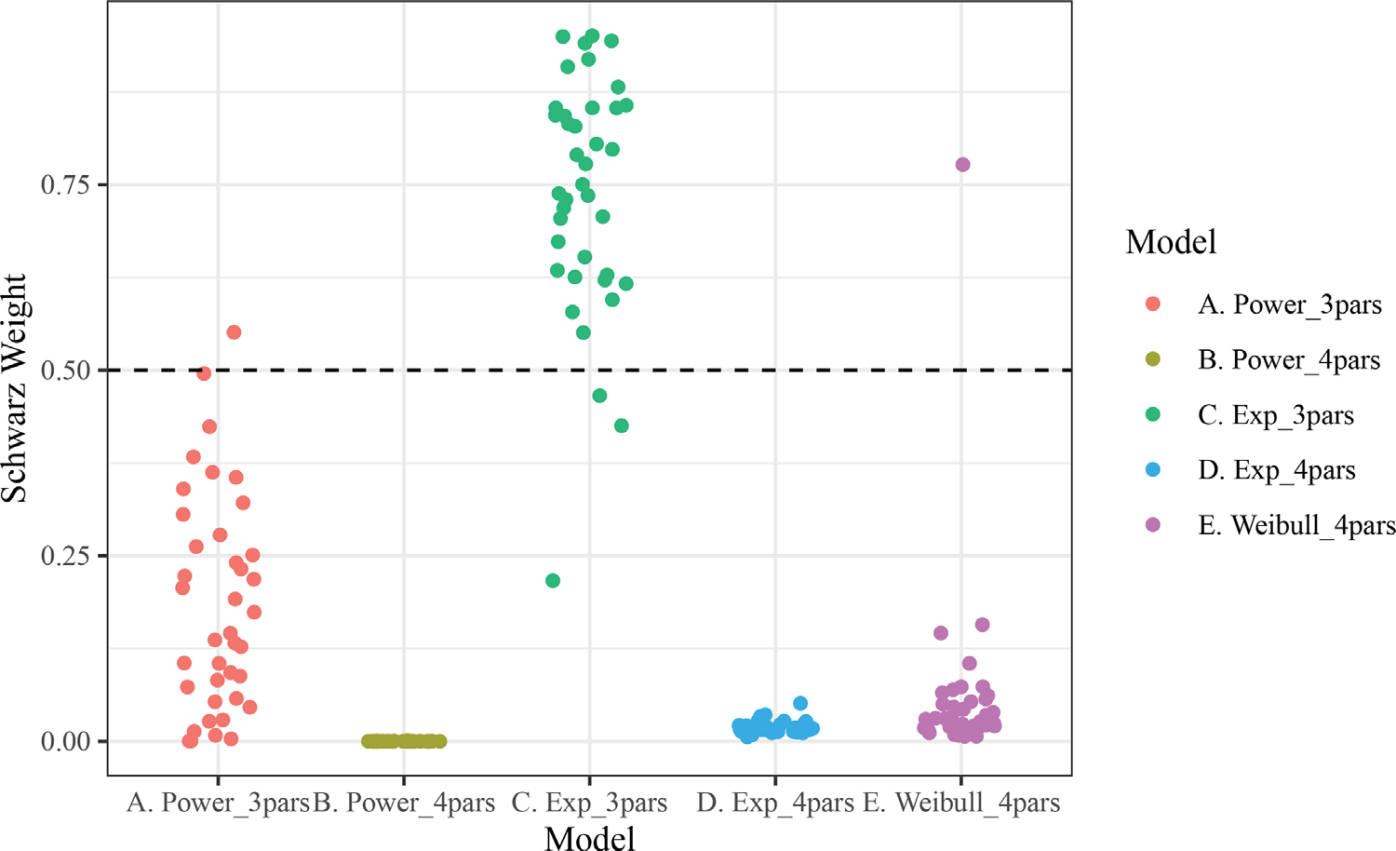
Texture oddball detection relative evidence for models, fit to both training and generalization trials, using each learning function. Each participant, in both difficulty conditions, has a weight (point) for each model. Weights are normalized BIC within participants and across models. All points above the dashed line indicate that, for a given subject, the model received more weight than all other models combined.

The primary analysis of interest involved comparisons of the relative likelihood for each model (i.e., Schwarz weights). Consistent with previous work examining individual-level learning (Dosher & Lu, 2007; Heathcote et al., 2000), the simplest function from the exponential family, the three-parameter exponential, was the best-fitting model to the vast majority of texture oddball detection participants (see Figure 5). The weights of the three-parameter exponential did not vary by difficulty (easy best fit = 95% of participants; difficult best fit = 89.5% of participants; coefficient of difficulty predicting three-parameter exponential weights in a bootstrapped robust linear model, *b* = −0.023, CI [−0.13, 0.065], Δ*R*^2^_oos_ = −0.0157).

**Figure 6.**
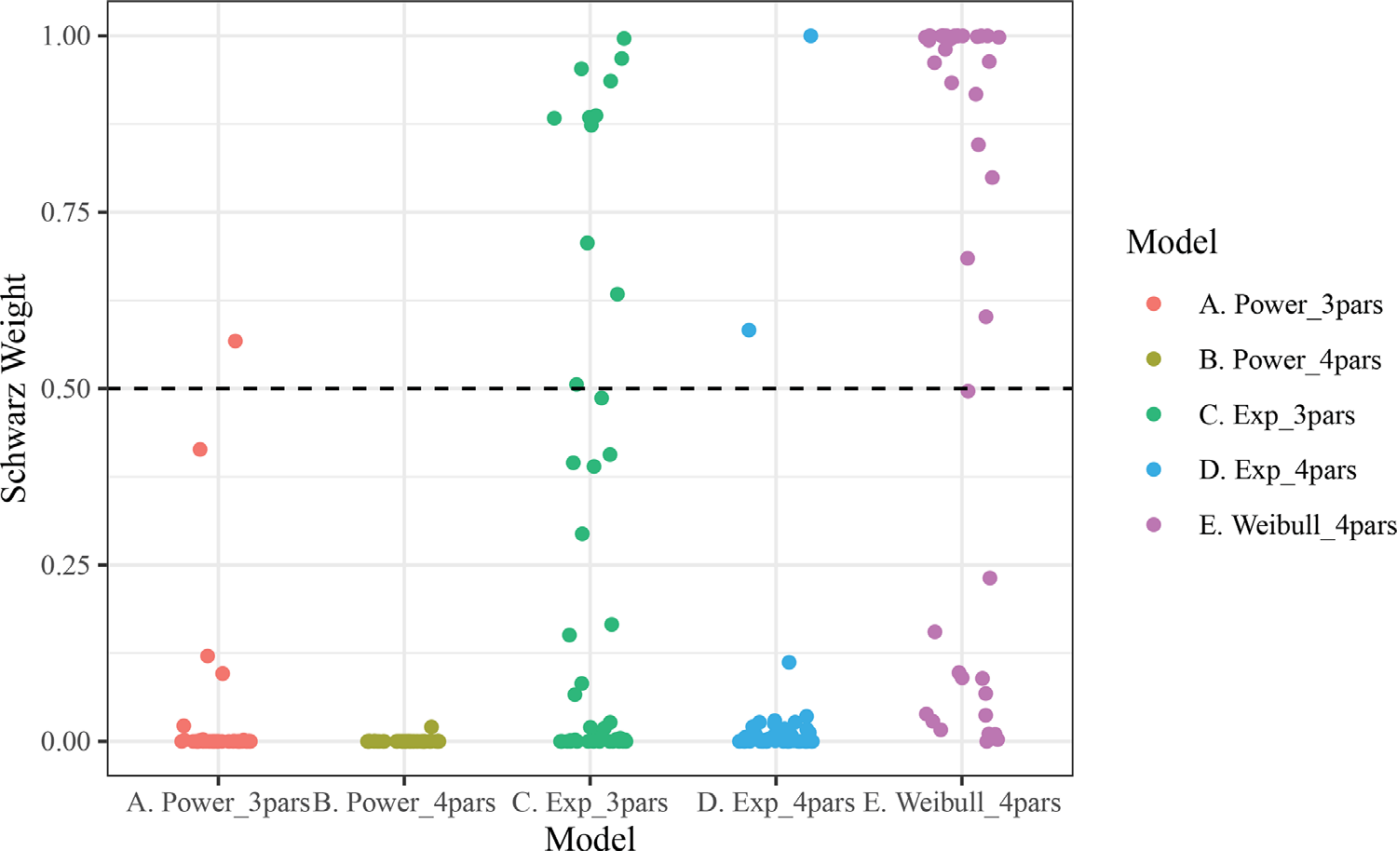
Dot-motion direction discrimination relative evidence for models, fit to both training and generalization trials, using each learning function. Each participant, from each difficulty condition, has a weight (point) for each model. Weights are normalized BIC within participants and across models. All points above the dashed line indicate that, for a given subject, the model received more weight than all other models combined.

Learning in dot-motion direction discrimination meanwhile tended to be best fit by the four-parameter Weibull model, an augmentation of the exponential function (see Figure 6). The weights of the Weibull models did not vary by difficulty (easy best fit = 69.6% of participants; difficult best fit = 60% of participants; coefficient of difficulty predicting Weibull weights in a robust linear model, *b* = −0.06, CI [−0.43,0.23], Δ*R*^2^_oos_ = −0.017). Of the participants whose learning was best characterized by the Weibull function, 93.1% had shape parameters over 0, indicating the benefits of the extra parameter were derived from fitting learning with a slow start (i.e., sigmoid shape and increasing hazard rate, as shown in Figure 4). Nearly all of participants who were not best fit by the Weibull function were instead best fit by the three-parameter exponential function (25.6% of all participants), which is equivalent to a Weibull with a shape parameter of 0.

Similar results were found when models were fit only to the trials in the training set (i.e., excluding the generalization trials). In this case, in the dot-motion task, 60% of participants were best fit by the Weibull model, while 35% were best fit by the three-parameter exponential model. Likewise, in the oddball detection task, 92% were best fit by the three-parameter exponential model.

In addition to our primary BIC analyses reported above, we conducted model comparisons using a similar cross-validation procedure as described above (i.e., fitting models to random 80% subsamples and assessing change in model log-likelihood when evaluating the fit parameters on the held-out 20% of data; the by-participant median out-of-sample change in log-likelihood was used as an index of model performance). Due to computational limitations, we completed this procedure only 50 times for each combination of participants and candidate functions, which limits the precision of this measure (i.e., making within-participant comparisons less robust). Nonetheless, the overall results were the same as the BIC analyses reported above. In the oddball detection task, the three-parameter exponential function led to fits with the best average out-of-sample performance, whereas in the dot-motion task, the Weibull function's fits gave the best average out-of-sample performance.

As a test of the implications of our by-subject models, we conducted a comparison analysis in which accuracy or d-prime had been averaged across participants for each trial. In this case, the three-parameter exponential function was the best-fitting model to the texture detection data (Schwarz weight: .949), while the four-parameter power function was the best-fitting to the dot-motion discrimination data (Schwarz weight: .999). In the dot-motion discrimination data, then, we recapitulated previous findings that aggregating perceptual learning data across participants can lead to a completely different functional form than the function form taken by individual learners' trajectories (Dosher & Lu, 2007). We note that these models, although implementing a more traditional aggregation-based approach, have a host of theoretical weaknesses and as such would not be recommended for future researchers to implement (Dosher & Lu, 2007; Heathcote et al., 2000; Kattner, Cochrane, & Green, 2017).

### Results: Form of generalization

We next turned to adjudicating between forms of generalization. Previous work has typically utilized quite short (i.e., single blocks) generalization tests and/or has employed significant aggregation of generalization task data. As such, it is unclear, when generalization has been found, whether the generalization is present from the very first trial of the new task (i.e., “immediate transfer”) or if, instead, the generalization manifests as faster learning of the generalization task. To adjudicate between these possibilities, we proceeded by testing the parameter distributions of generalization start and rate (themselves each parameterized as offsets from the associated parameters in initial learning; see Figure 3). We used fit parameters from the overall best-fitting models for each task (i.e., dot-motion task Weibull and oddball detection task three-parameter exponential). Due to the explicitly one-sided nature of the tests, we used 50,000 resamples with replacement of the mean of participants' parameters to estimate the bootstrapped one-tailed lower or upper 95% bound (i.e., generalization would only be due to decreases in threshold, increases in d-prime, or decreases in the amount of time taken to learn; changes in the opposite direction could not be interpreted as successful generalization). Due to the possibility of differences in generalization due to task difficulty (see also next section for explicit comparisons across difficulty levels), we tested each task and each difficulty level independently.

**Figure 7.**
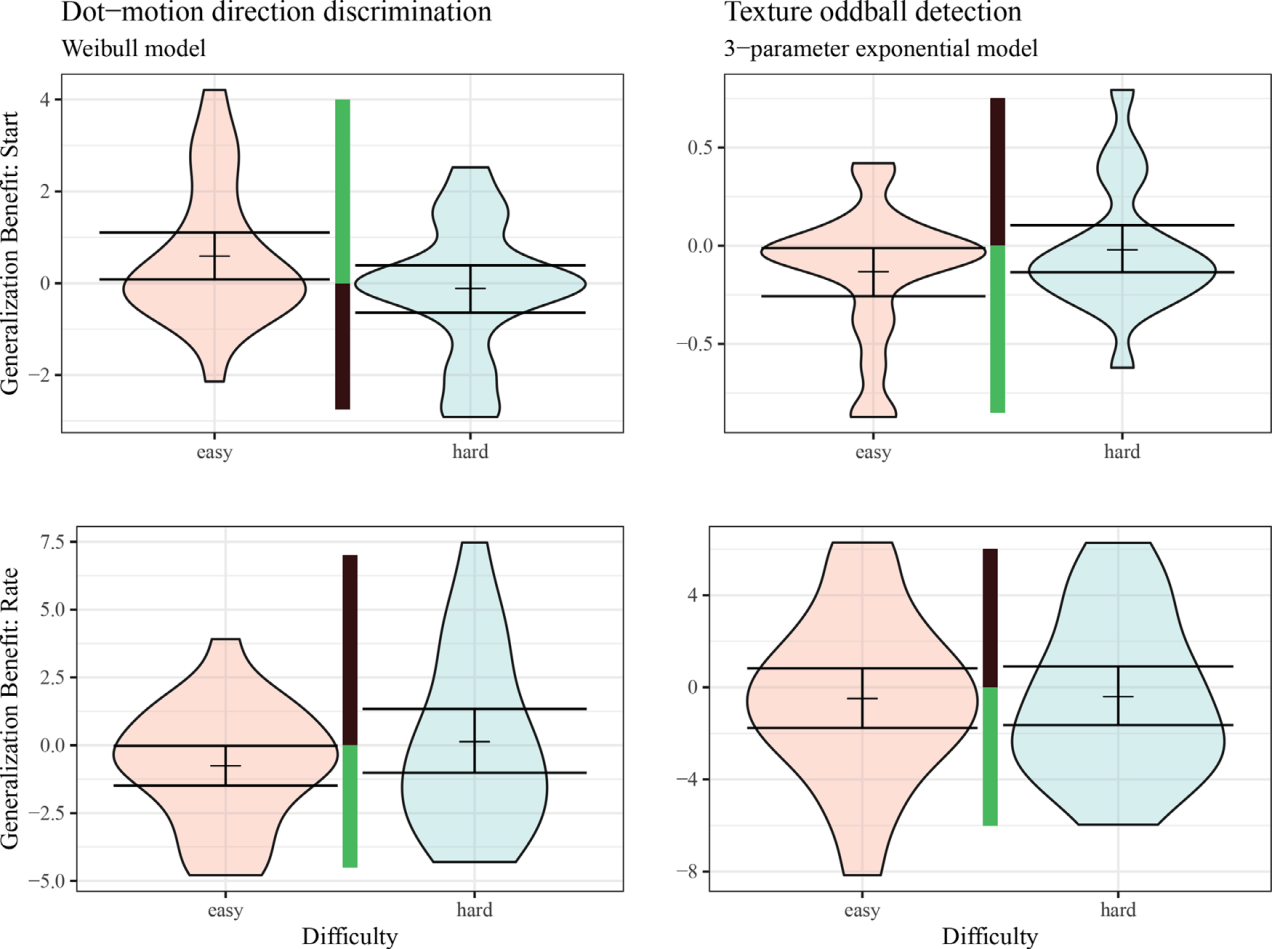
Differences between generalization parameters and training parameters. The most frequently best-fitting model was used for each task. Central guide bars show, in green, the direction of generalization benefits (i.e., either positive or negative values, depending on the particular parameter—i.e., for start values: “generalization,” meaning better performance at the start of the generalization block than was observed at the start of the initial block, is indicated by higher values of accuracy for the dot-motion task and lower values of threshold for the texture task). Shaded area indicates smoothed density. Lines indicate mean and bootstrapped 90% CI (i.e., reflecting one-sided tests from Results).

Starting texture oddball detection generalization thresholds of participants in the easier condition were reliably lower than their initial learning starting thresholds (mean = −0.13, CI_upper_ = −0.02), while the difficult group did not demonstrate reliably lower thresholds at the start of generalization (mean = −0.02, CI_upper_ = 0.1; see Figure 7). No reliable pattern of generalization due to a decrease in time taken to learn was evident in either difficulty condition (rate parameter difference: *easy* mean = −0.48, CI_upper_ = 0.8; *hard* mean = −0.4, CI_upper_ = 0.9). Thus, the only evidence for any generalization was in the form of immediate transfer.

The results of the dot-motion discrimination task were similar to the texture oddball detection task. Participants in the easier condition had dot-motion d-prime scores at the start of the generalization task that were reliably higher than their d-prime at the beginning of training (mean = 0.59, CI_lower_ = 0.09; see Figure 7). In contrast, starting d-prime was no higher in the generalization than in initial learning for participants in the difficult condition (mean = −0.11, CI_lower_ = −0.64). The easier condition but not the difficult condition demonstrated learning to learn, via decreases in the time taken to learn, in generalization (rate parameter difference: *easy* mean = −0.75, CI_upper_ = −0.01; *hard* mean = 0.13, CI_upper_ = 1.33). However, due to concerns that the Weibull rate parameters may be biased by certain patterns in the Weibull shape parameters (shared across initial learning and generalization) as well as due to the minority support for the three-parameter exponential model in the motion discrimination task, we tested possible dot-motion generalization in the rate parameter of the three-parameter exponential. In these analyses, we observed null effects when comparing generalization and learning rates in the three-parameter exponential model (rate parameter difference: *easy* mean = 0.00, CI_upper_ = 0.91; *hard* mean = 0.56, CI_upper_ = 1.75). As a whole, then, there was greater support for an immediate-transfer mechanism of generalization than one involving learning to learn.

### Results: Generalization by difficulty interactions

Given the lack of differences in functional form by difficulty, using each experiment's most common best-fit models (i.e., three-parameter exponential or Weibull), we next assessed the degree to which training task difficulty moderated generalization. Figure 7 shows that easy conditions did appear to lead to greater generalization of learning in the form of starting ability. This is consistent with previous reports (e.g., Ahissar & Hochstein, 1997; Wang et al., 2013) while adding information about the time course of generalization.

In each task, we tested for the effect of difficulty on starting generalization in two ways. In the first method, we fit a bootstrapped robust linear model predicting the generalization starting parameter using difficulty condition while controlling for each participants' training start parameters. In the second, we normalized the magnitude of the generalization start parameter by dividing it by the difference between a participant's asymptotic level and their training start. We then fit a bootstrapped robust linear model to predict this proportional benefit of generalization using difficulty condition.

In oddball texture detection, training on the easy condition generalized more than that training on the difficult condition (*b* = 0.11, CI [0.003, 0.2], Δ*R*^2^_oos_ = 0.0988). However, this effect was lost when the generalization benefit was calculated as a proportion of total learning (*b* = −0.12, CI [−0.9,0.63], Δ*R*^2^_oos_ = −0.0122). Likewise, when examining dot-motion orientation discrimination parameters directly, the easy condition generalized more than the difficult condition (*b* = −1.1, CI [−2, −0.24], Δ*R*^2^_oos_ = 0.2106). When calculating dot-motion generalization benefit as a proportion of learning, there was no difference between difficulties (*b* = 0.03, CI [−0.77,0.69], Δ*R*^2^_oos_ = −0.0116).

We next used bootstrapped robust linear models to predict generalization rate using difficulty condition while controlling for training rate. There were no differences in generalization of learning rate between difficulty conditions for either learning paradigm. Clearly, to the extent that differences in generalization due to difficulty were evident, these group differences were due to immediate benefits in performance rather than differences in the rate of generalization.

### Recovery analyses

Newell and Rosenbloom (1981) famously described “curve fitting” of learning as a “black art.” Despite 40 years of computational and empirical advances that have massively enhanced our abilities in this space, we nonetheless remain fundamentally limited by the basic mathematical facts that lead to models' ability to imitate one another. To determine the extent to which our models were empirically separable using our analytical methods, we conducted a recovery analysis. Each participant had five sets of fit model parameters from the primary analyses reported above. From those each of those model's parameters, we simulated new data sets to test the ability of the true generative model to fit the simulated data better than the other models.

In these analyses, the primary measure of interest was the extent to which the two three-parameter models were distinct from one another. This has a theoretical basis, as the family-level distinction has the deepest implications for the field of perceptual learning, but it also has a practical basis. Participants' parameters in their four-parameter models, each of which was nested within a three-parameter model, may not have provided a learning trajectory that was truly separable from the simpler three-parameter model. In this case, the likelihood penalty introduced in the BIC calculation would lead to the simpler model being chosen even when the true generative model was more complex. Because of this, we report here only the ability to recover the three-parameter power or exponential models. Full recovery analyses with every combination of models are reported in the Supplementary Material.

We simulated 40 new data sets from each participant's parameters for their dot-motion discrimination and texture oddball detection three-parameter models. The texture oddball detection accuracies were simulated from a Bernoulli distribution with the models' by-trial fit probabilities. The dot-motion discrimination model fit d-primes were first converted to accuracy probability values by assuming zero bias (i.e., *p*(*hit*) = *p*(*correct rejection*)), and by-trial accuracies were simulated from a Bernoulli distribution with these probabilities and randomly distributed “same-stimuli” and “different-stimuli” trials.

Given simulated data from each model, we then fit both three-parameter models to the data using the same methods as the primary analyses. One change was made, namely, that the number of optimization runs was only 200, or 10% of the primary analyses' number of optimization runs. This was due to the time constraints of running a very large number of models; we believe the reduction in optimization runs would not appreciably reduce the quality of our estimates.

Across the 40 simulations from the oddball detection data, the by-participant (across-simulation) recovery of learning models was not strong (mean within-participant d-prime = 0.372). In addition, 38.5% of participants had a bias toward the power model (within these, mean bias = 0.117) while 48.7% of participants had a bias toward the exponential model (within these, mean bias = 0.155). In contrast, by-participant recovery of dot-motion was better (mean within-participant d-prime = .652); 14% of participants had a bias toward the power model (within these, mean bias = 0.065) while 81.4% of participants had a bias toward the exponential model (within these, mean bias = .305). In combination, these analyses indicate that the dot-motion direction discrimination results are likely to be more robust than the results of the oddball detection task. In the oddball texture detection learning, then, the strength of our inferences is particularly limited by a likely upper bound on the ability to adjudicate between models.

The limitations indicated by the model recoverability results in both tasks demonstrate that, at the level of individual learners or small sample sizes, adjudicating between models may be nearly impossible. This provides further reason to use large sample sizes such as ours (relative to much perceptual learning work) to detect population-level trends in the best-fitting models to perceptual learning data. See the Supplementary Material for further recovery analysis results.

## Discussion

Here, in two different perceptual learning experiments, we examined the best-fitting functional form of learning on a by-trial and by-participant scale. Overall, functions coming from the exponential family provided the best trial-level fits to almost all participants' learning. In an oddball detection task, improvements were overwhelmingly best characterized by a simple three-parameter exponential model of change. Meanwhile, in a dot-motion direction discrimination task, improvements in d-prime were best characterized by a Weibull or a three-parameter exponential function. Because the three-parameter exponential function is nested within the Weibull function (i.e., the latter is an extension of the former with one extra parameter), both experiments repudiate the “power law of learning” (Newell & Rosenbloom, 1981) and corroborate evidence for exponential functions over power functions (Dosher & Lu, 2007). The dominance of the exponential family was uniform across levels of task difficulty, thereby precluding the possibility that difficulty-related variations in learning may lead to distinct shapes. This thus provides a unique converging data point on the issue, as previous research has often utilized adaptive algorithms to generate stimulus difficulty (i.e., has attempted to match subjective difficulty across participants, whereas here we had versions that differed in stimulus strength constants). The evidence for the exponential family was independent of whether comparisons were being made between models fit to only training data or to training data plus generalization data.

The nested nature of the three-parameter exponential function within the Weibull function also carries the implication that all models utilizing the three-parameter exponential function would be equally well fit by the Weibull function. That is, the three-parameter exponential function is a special case of the Weibull function, and as such, all learning best fit by the three-parameter exponential function can likewise be seen as being characterized by a special case of the Weibull. Model comparisons between these two models served simply to test whether the extra “start speed” (i.e., change in hazard rate or “shape”) parameter of the Weibull function was statistically justified through a sufficient reduction in model error. The results of the dot-motion learning experiment demonstrate the utility of comparing these nested models to assess the appropriateness of the shape parameter. In other words, although all learning was best fit by a Weibull function, some of these Weibull functions could be reduced to the three-parameter exponential function without compromising model fit.

Support for an exponential family of change implicates a mechanistically simple process of learning in each paradigm. The novelty of the continuous-time approach to learning thereby corroborated earlier work in perceptual learning that supported a unitary process of change underlying learning (Dosher & Lu, 2007), as compared to averaged or cascading processes (e.g., Ahissar & Hochstein, 2004). While oddball detection learning was best characterized as arising from learning a constant proportion on each learning event (i.e., constant hazard rate), in many participants, dot-motion direction learning was better characterized by including a parameter that allowed the hazard rate to either increase or decrease. The overwhelming majority of these latter participants displayed an acceleration in learning (i.e., a sigmoid function). An increasing hazard rate appears to indicate an interaction between a simple exponential mechanism of learning and a second mechanism that prevented immediately constant learning and thereby slowed down initial learning (e.g., a “releasing” mechanism). This mechanistic explanation remains speculation, however, and the current results provide the impetus for a more thorough investigation of the potential dissociations or interactions between simple learning processes and modulatory mechanisms that may inhibit the initial learning rate. It may be possible to identify distinct processes, for example, by using convergent methods (i.e., higher-dimensional data). In addition, future work should explore the experimental conditions and the performance indices for which initial learning is slowed down in comparison to conditions under which the hazard rate is constant.

Regardless of the precise mechanistic underpinnings of the Weibull learning observed here, it stands in stark contrast to any power-family model. While power-family models implement a decreasing hazard rate, the increasing hazard rate allowed by the Weibull function or any other sigmoid function (and observed in many participants here) is quite the opposite. The lack of a decreasing hazard rate also puts the present results in contrast to a body of work that posits a fast early phase of perceptual learning that is followed by a slower phase (e.g., Ahissar & Hochstein, 1997; Newell et al., 2009). Due to the scarcity of previously reported by-trial methods in perceptual learning, as well as a lack of direct comparisons between accelerating and nonaccelerating functions, we cannot know the extent to which our results are anomalous. Indeed, previous work has used comparisons that explicitly compare functions that allow a constant or decelerating hazard rate, or a combination of the two (e.g., using an APEX function, which itself subsumes a power and an exponential function; Dosher & Lu, 2007; Heathcote et al., 2000). Nonetheless, our results uniformly lack support for a decelerating hazard rate, such as that which could be instantiated by a power function or a “fast then slow learning” model.

After comparisons of learning functions, we tested the possible forms of generalization in each of our tasks. In both perceptual learning paradigms, there was evidence for generalization as immediate transfer (i.e., improved starting performance), while evidence for generalization via changes in learning rate was inconsistent. Furthermore, and consistent with previous research (Ahissar & Hochstein, 1997, 2000; Ball & Sekuler, 1987; Jeter et al., 2009; Wang et al., 2013), the degree of observed generalization differed as a function of task difficulty. Greater generalization was found in both tasks' easy conditions relative to the associated difficult conditions. However, here it is important to note that because our methods confounded training difficulty and generalization difficulty, it is not possible to ascertain whether the difficulty of the training task (e.g., as argued in Ahissar & Hochstein, 1997) or the difficulty of the generalization task (e.g., as argued in Jeter et al., 2009) is the driving factor. Our approach also uncovered the fact that the between-subjects difficulty-related effect was not robust when generalization was calculated as a relative rather than absolute benefit, indicating that overall learnability differences between easy and difficult tasks may determine performance in ways that may imitate disproportionate benefits of easy-task training.

To make this last point more concrete using dot-motion perceptual learning as an example, cross-participant d-prime increased from start to asymptote. Although the easy condition's first generalization trials improved when compared directly to their initial performance, the difficult condition's generalization was reliably smaller. However, the fact that the *overall amount* of learning in the easy condition was greater than the difficult conditions means that the definition of generalization itself is not clear a priori. Although the benefits of the easy condition relative to the difficult condition were clear when considering only starting parameters (keeping in mind that the two curves shared an asymptote), there was no such effect when considering the overall amount learned in the different difficulty conditions.

While the results reported here provide uniform evidence for the exponential family of learning, as well as show novel support for a function that can take a sigmoid shape in perceptual improvements, we do recognize that our inferences are limited by the relatively short timescale of the training. Two days and 1,200 trials of training would be substantial in many domains of learning, but perceptual learning studies have frequently implemented an order of magnitude more days and trials (e.g., Ahissar & Hochstein, 1993; Cochrane et al., 2019; but for shorter timescales of training, see Fiorentini & Berardi, 1980; Mednick et al., 2002; Poggio et al., 1992). While the support for the exponential function indicates that the timescale may have minimal impact on our results (i.e., because of the constant hazard rate, an exponential function is translation invariant; Heathcote et al., 2000), future studies of longer-timescale perceptual learning are warranted by the increases in power and the possible process-level heterogeneity in longer-term improvements (Shibata et al., 2014).

In our comparisons of the functional forms of learning, we necessarily restricted our approach to a small number of models that were drawn from the exponential or power families. Many other possible forms of learning would be possible to test, however. For instance, previous work has utilized the APEX function, a combination of power and exponential functions. The APEX function is very useful for conducting nested model comparisons (i.e., because it subsumes the simpler exponential and power functions). However, our model comparison method did not require nested models, thereby providing the ability for us to focus our comparisons on functions that fell more strictly within one family or the other. Still other investigations of functional forms in learning have addressed forms with more mathematical (and implied process-level) complexity (e.g., sequential exponential functions; Dosher & Lu, 2007; sequential power functions; Donner & Hardy, 2015; “phases” of learning; Tenison & Anderson, 2016). In perceptual learning, there has not been evidence supporting this proposition (Dosher & Lu, 2007). Nonetheless, while a wider variety of functional forms may be reasonable to investigate in future work, our limited amount of data per participant has constrained our ability to test these more complex models. Alternative models could also include characterizations of learning that do not impose particular functional forms on trajectories of performance (e.g., reinforcement learning or other dynamic models; Law & Gold, 2009; Roy et al., 2021); however, comparisons with these approaches are outside the scope of the present work.

## Conclusions

The present study involved two perceptual learning experiments. Continuous-time modeling provided uniform support for unitary exponential learning processes, with a possible need to account for a second process modulating the initial speed of learning (possibly related to releasing the “brakes” on learning; Bavelier et al., 2010). There was no support for the use of power functions to understand learning processes, leading to the recommendation to use the three-parameter exponential function or the more flexible Weibull function rather than functions from the power family in order to most appropriately characterize the changes in processes associated with within-individual perceptual learning. The extent to which these two exponential-family approaches should both be fit, as well as model comparison used to either justify or reject the extra parameter associated with the Weibull function, will be constrained by the quantity and quality of behavioral data. In experiments with sparse or noisy data, it is likely that the flexibility of the Weibull function will lead to overfitting. The evidence in favor of the exponential family across behavioral paradigms and task difficulties, as well as when incorporating both initial training and later generalization, provides an empirical foundation for the use of this family.

## Supplementary Material

Supplement 1
