# Supplementary material for: Assessing the functions underlying learning using by-trial and by-participant models: Evidence from two visual perceptual learning paradigms
Source: J Vis. 2021 Dec 14;21(13):5. doi: 10.1167/jov.21.13.5 (PMC8684311; doi:10.1167/jov.21.13.5)
Supplement: Supplement 1 [file jovi-21-13-5_s001.pdf]

Supplementary Material

Supplemental methods

Dot-motion direction discrimination

All participants completed their blocks alone in a dimly-lit sound-attenuated room in the psychology lab of C.S.G. Instructions were provided in the form of a piece of paper with a schematic explanation of the task. This visual explanation was accompanied by a verbal explanation by an experimenter.

On each day 4 blocks of 200 trials were run, with short breaks between each block. Each block took between 12 and 15 minutes to complete. Stimulus order was fully randomized within blocks. Auditory feedback was provided after each trial in the form of a high-pitched beep for a "correct" response and a low-pitched beep for an "incorrect" response. The first 6 blocks used a reference angle of 40 degrees, with the last 2 blocks utilizing a reference angle of 130 degrees. Before the first and seventh blocks the participant completed 8 very easy trials (12 degree offset between the reference angle and the stimulus angle) in order to ensure understanding of the task.

All stimuli were presented centrally, with free fixation. The difficulty manipulation used offsets of 4 (easy) or 8 (hard) degrees, following Wang et al. (2013). This meant that each stimulus was offset either 2 degrees (easy) or 4 degrees (difficulty) either clockwise or counterclockwise from the reference angle.

Texture oddball detection

All participants completed their blocks alone in a dimly-lit sound-attenuated room in the psychology lab of C.S.G. Instructions were provided in the form of a piece of paper with a schematic explanation of the task. This visual explanation was accompanied by a verbal explanation by an experimenter.

On each day 4 blocks of 210 trials were run, with short breaks between each block. Each block took between 12 and 15 minutes to complete. Stimulus order was fully randomized within blocks. Auditory feedback was provided after each trial in the form of a high-pitched beep for a "correct" response and a low-pitched beep for an "incorrect" response. The first 6 blocks used a reference angle of 16 degrees, with the last 2 blocks utilizing a reference angle of 106 degrees. Before the first and seventh blocks the participant completed 8 very easy trials (SOA of .24 and .48) in order to ensure understanding of the task.

All stimuli were presented centrally, with free fixation. The difficulty manipulation used oddball offsets of 16 (easy) or 30 (hard) degrees, following Ahissar & Hochstein, (1993).

Functional Forms

This work used **TEfits** version 00.77.12 (Cochrane, 2020). The formulas, from **TEfits** as well as in simplified formats, for the functional forms were as follows:

3-parameter power (formula)	$start + (asymptote - start) \times time^{rate}$
3-parameter power (model implementation)	~ ((pAsym) + ((pStart_0 + pStart_isTransfer * isTransfer) - (pAsym)) * (totalTrialNum - 0)^(log(0.25)/log(2^pRate_0 + pRate_isTransfer * isTransfer)))
4-parameter power (formula)	$start + (asymptote - start) \times (time + previousTime)^{rate} \times \frac{1}{previousTime^{rate}}$
4-parameter power (model implementation)	~ ((pAsym) + ((pStart_0 + pStart_isTransfer * isTransfer) - (pAsym)) * (((totalTrialNum - 0) + pPrevTime)^(log(0.25)/log(2^(pRate_0 + pRate_isTransfer * isTransfer)))) * (1/((pPrevTime + 1)^(log(0.25)/log(2^(pRate_0 + pRate_isTransfer * isTransfer))))))
3-parameter exponential (formula)	$start + (asymptote - start)^{time \times rate}$
3-parameter exponential (model implementation)	~ ((pAsym) + ((pStart_0 + pStart_isTransfer * isTransfer) - (pAsym)) * 2^((1 - totalTrialNum)/(2^(pRate_0 + pRate_isTransfer * isTransfer))))
4-parameter exponential (formula)	$start + .5 \times (asymptote - start)^{time \times rate_1} + .5 \times (asymptote - start)^{time \times rate_2}$
4-parameter exponential (model implementation)	~ ((pAsym) + ((pStart_0 + pStart_isTransfer * isTransfer) - (pAsym)) * 0.5 * 2^((1 - totalTrialNum)/(2^(pRateA_0 + pRateA_isTransfer * isTransfer))) + ((pStart_0 + pStart_isTransfer * isTransfer) - (pAsym)) * 0.5 * 2^((1 - totalTrialNum)/(2^(pRateB))))
Weibull (formula)	$start + (asymptote - start)^{(time \times rate)^{shape}}$
Weibull (model implementation)	~ ((pAsym) + ((pStart_0 + pStart_isTransfer * isTransfer) - (pAsym)) * 2^(-((totalTrialNum - 1)/(2^(pRate_0 + pRate_isTransfer * isTransfer)))^(2^pShape)))

These formulas predicted either d-prime (in the case of dot-motion discrimination) or 75% accuracy threshold (in the case of texture oddball detection). Note that constants (e.g., `2` as the base of the exponents or the `log(.25)` present in the power functions) exist to make parameter values themselves more interpretable, and have no influence on the overall models' goodness-of-fit. The same can be said for most aspects of the parameterizations; **TEfits** prioritizes interpretability of parameters, with some sacrifice to the clarity of model formulas. The generalization parameters, `pStart_isTransfer` and `pStart_isTransfer`, were associated with the binary (0 or 1) variable `isTransfer`.

Model code

Dot-motion direction discrimination model code (**TEfits** package)

```
nTries <- 2E3
nBoot <- 50

 m_tef_exp3 <- TEfitAll(motDat[,c('dPrime',"totalTrialNum","isTransfer")],
            errFun='ols',changeFun='expo', covarTerms=list(pAsym=F),
            bootPars=list(nBoots=nBoot,bootPercent=.8),
            control = tef_control(suppressWarnings=T,nTries=nTries,y_lim=c(0,5)),
            groupingVar=motDat$subID,groupingVarName = 'subID')

 m_tef_exp4 <- TEfitAll(motDat[,c('dPrime',"totalTrialNum","isTransfer")],
            errFun='ols',changeFun='expo_double', covarTerms=list(pAsym=F),
            bootPars=list(nBoots=nBoot,bootPercent=.8),
            control = tef_control(suppressWarnings=T,nTries=nTries,y_lim=c(0,5)),
            groupingVar=motDat$subID,groupingVarName = 'subID')

 m_tef_pow3 <- TEfitAll(motDat[,c('dPrime',"totalTrialNum","isTransfer")],
            errFun='ols',changeFun='power', covarTerms=list(pAsym=F),
            bootPars=list(nBoots=nBoot,bootPercent=.8),
            control = tef_control(suppressWarnings=T,nTries=nTries,y_lim=c(0,5)),
            groupingVar=motDat$subID,groupingVarName = 'subID')

 m_tef_pow4 <- TEfitAll(motDat[,c('dPrime',"totalTrialNum","isTransfer")],
```

```
            errFun='ols',changeFun='power4', covarTerms=list(pAsym=F,pPrevTime=F),
            bootPars=list(nBoots=nBoot,bootPercent=.8),
            control = tef_control(suppressWarnings=T,nTries=nTries,y_lim=c(0,5)),
            groupingVar=motDat$subID,groupingVarName = 'subID')

m_tef_weib <- TEfitAll(motDat[,c('dPrime',"totalTrialNum","isTransfer")],
            errFun='ols',changeFun='weibull', covarTerms=list(pAsym=F),
            bootPars=list(nBoots=nBoot,bootPercent=.8),
            control = tef_control(suppressWarnings=T,nTries=nTries,y_lim=c(0,5),
                    shape_lim=c(-2,2)),
            groupingVar=motDat$subID,groupingVarName = 'subID')
```

Notes:

- ***nTries*** *indicates the number of attempted optimization runs, to minimize error with the optim() function in R initialized at random starting points.*
- *Due to the superior performance of the Weibull function in preliminary analyses, there was some concern that extreme flexibility may have allowed it to take the fit trajectory to implausible values. As such, we restricted the Weibull shape parameter to [-2,2]; the Weibull function still was the best fit in many cases.*
- *As explained in the main text, d-prime was bounded at [0,5] due to this being the entire range of plausible performance.*

Texture oddball detection model code (**TEfits** package)

```
nTries <- 2E3
nBoot <- 50

m_tef_exp3 <- TEfitAll(texDat[,c('Corr',"totalTrialNum","SOA","isTransfer")],
            errFun='bernoulli',linkFun = list(link='weibull',weibullX='SOA'),
            changeFun='expo',
            covarTerms=list(threshAsym=F),
            bootPars=list(nBoots=nBoot,bootPercent=.8),
            control = tef_control(suppressWarnings=T,nTries=nTries),
```

```
                groupingVar = texDat$subID,groupingVarName = 'subID')

m_tef_exp4 <- TEfitAll(texDat[,c('Corr',"totalTrialNum","SOA","isTransfer")],
                  errFun='bernoulli',linkFun = list(link='weibull',weibullX='SOA'),
               changeFun='expo_double',
               covarTerms=list(threshAsym=F),
                bootPars=list(nBoots=nBoot,bootPercent=.8),
               control = tef_control(suppressWarnings=T,nTries=nTries),
               groupingVar = texDat$subID,groupingVarName = 'subID')

m_tef_pow3 <- TEfitAll(texDat[,c('Corr',"totalTrialNum","SOA","isTransfer")],
                  errFun='bernoulli',linkFun = list(link='weibull',weibullX='SOA'),
               changeFun='power',
               covarTerms=list(threshAsym=F),
               bootPars=list(nBoots=nBoot,bootPercent=.8),
               control = tef_control(suppressWarnings=T,nTries=nTries),
               groupingVar = texDat$subID,groupingVarName = 'subID')

m_tef_pow4 <- TEfitAll(texDat[,c('Corr',"totalTrialNum","SOA","isTransfer")],
                   errFun='bernoulli',linkFun = list(link='weibull',weibullX='SOA'),
                   changeFun='power4',
                   covarTerms=list(threshAsym=F,pPrevTime=F),
                   bootPars=list(nBoots=nBoot,bootPercent=.8),
                   control = tef_control(suppressWarnings=T,nTries=nTries),
                   groupingVar = texDat$subID,groupingVarName = 'subID')

m_tef_weib <- TEfitAll(texDat[,c('Corr',"totalTrialNum","SOA","isTransfer")],
                   errFun='bernoulli',linkFun = list(link='weibull',weibullX='SOA'),
                   changeFun='weibull',
                   covarTerms=list(threshAsym=F),
                   bootPars=list(nBoots=nBoot,bootPercent=.8),
                   control = tef_control(suppressWarnings=T,nTries=nTries),
```

```
              groupingVar = texDat$subID,groupingVarName = 'subID')
```

Notes:

- ***nTries** indicates the number of attempted optimization runs, to minimize error with the optim() function in R initialized at random starting points.*

Generalization measured as a time equivalent

An alternative measure of initial generalization magnitude was tested. By considering starting performance on the generalization trials, the threshold or d-prime could be mapped onto the learning trajectory from the initial training trials. We note, however, that this analysis discards any differences in learning rate between initial training and subsequent generalization. Additionally, we note that many participants have a "time equivalent" that would be negative, as many of participants did not demonstrate generalization (see Figure 7 for distributions of participants; the density of the distributions on the non-green side of zero would all have negative time equivalents). Rather than calculating negative time equivalents we treated all such cases as having time equivalents of 1 (i.e., starting generalization at the same place as starting initial training). However, given these very non-normal distributions, we chose to compare difficulty-related differences using non-parametric Wilcoxon tests. Using this method, in texture oddball detection, the easy-condition time equivalents (median = 348) were not significantly different than the difficult-condition time equivalents (median = 94; Wilcoxon Z = -1.69, p = .09). Likewise, in dot-motion direction discrimination, the easy-condition time equivalents (median = 63) were not significantly different than the difficult-condition time equivalents (median = 3; Wilcoxon Z = 1.10, p = .271).

Recovery Analyses

As explained in the main text, recovery analyses can be ambiguous when simulating and comparing parameters from nested models. Specifically, because several of our 4-parameter models can take exactly the same shapes as simpler 3-parameter models, it may be impossible to recover the more complex models when compared against the fits from simpler models.

With this caveat, we next report several measures of parameter recovery. Using the methods reported in the main text, we used 40 simulations from each model's parameters from each participant to compare models. The first measure we use is mean within-simulation d-prime. That is, within each simulation, and when comparing two models, each participant was either correctly categorized using a BIC criterion (a "hit" or "correct rejection") or incorrectly categorized (a "miss" or "false alarm"). The distinction between "hit" and "correct rejection" was arbitrarily assigned to one or the other of the

compared models. Given the vector of correct or incorrect categorizations we calculated a sensitivity measure for each simulation (d-prime) and a second measure, a bias term (c). We present each of these in tables below. Note that d-prime is symmetric across the diagonal, while c refers to the bias *for* the row-model and *against* the column-model (with c being sign-reversed across the diagonal). The last measure we present maintains more proximity to our primary analyses, in that we do not dichotomize "winning" and "losing" models. Instead, we used the BIC from every model fit as a predicted variable in linear mixed-effects models in which a dichotomous model type variable (e.g., 3-parameter power function vs. Weibull function) was a fixed effect and by-participant random intercepts and model type slopes were included (e.g., *BIC~modelType+(modelType|participantID)*). The by-participant random effects were appropriate because we included all simulations' BICs in these analyses. We then used the T values of the model type fixed effect as a measure of the reliability of our recovery. We recognize that with an arbitrarily large number of simulations these T values would likewise become arbitrarily large (positively or negatively), however, we believe that the relative magnitudes of these different T values with the present number of simulations was very instructive regarding the robustness of our model recovery. The tables with these T values, unlike the d-prime and c tables, are not symmetric. Instead, the row indicates the true generative model for the simulation, and the column indicates the T value of the BIC coefficient for that model. Positive values indicate that the true generative model tended to have lower BIC (i.e., "win"), whereas negative values indicate that the incorrect model tended to have the lower BIC.

In summary, these analyses corroborate the brief recovery analyses reported in the main text. While the recovery of functional forms in texture oddball detection learning was highly inconsistent, the recovery of dot-motion direction discrimination functional forms were somewhat more consistent.

	exp3	*exp4*	*pow3*	*pow4*	*weib*
exp3		0.047	0.37	0.055	-0.01
exp4	0.047		0.129	-0.047	-0.018
pow3	0.37	0.129		0.071	0.07
pow4	0.055	-0.047	0.071		-0.021
weib	-0.01	-0.018	0.07	-0.021	

Table A1. Texture oddball detection recovery d-primes for all models. D-primes were calculated from the pairwise model recovery across participants and within each simulation, and the average values are presented here.

	exp3	*exp4*	*pow3*	*pow4*	*weib*

exp3		0.158	0.03	0.326	0.155
exp4	-0.158		-0.142	0.173	-0.024
pow3	-0.03	0.142		0.303	0.15
pow4	-0.326	-0.173	-0.303		-0.208
weib	-0.155	0.024	-0.15	0.208	

Table A2. Texture oddball detection recovery bias (c) for all models. Numbers refer to the bias for the row-model and against the column-model (with c being sign-reversed across the diagonal).

	exp3	*exp4*	*pow3*	*pow4*	*weib*
exp3		1.229	1.011	2.124	0.878
exp4	-0.682		-0.315	1.01	-0.251
pow3	0.572	1.13		2.046	1.092
pow4	-1.966	-1.319	-1.572		-1.118
weib	-0.873	0.101	-0.3	1.339	

Table A3. Texture oddball detection recovery T values for all models

	exp3	*exp4*	*pow3*	*pow4*	*weib*
exp3		0.097	0.517	0.415	0.117
exp4	0.097		0.266	0.37	0.066
pow3	0.517	0.266		0.074	0.428
pow4	0.415	0.37	0.074		0.484
weib	0.117	0.066	0.428	0.484	

Table A4. Dot-motion direction discrimination recovery d-primes for all models. D-primes were calculated from the pairwise model recovery across participants and within each simulation, and the average values are presented here.

	exp3	*exp4*	*pow3*	*pow4*	*weib*
exp3		0.051	0.223	0.47	-0.026
exp4	-0.051		0.161	0.403	-0.065
pow3	-0.223	-0.161		0.236	-0.27
pow4	-0.47	-0.403	-0.236		-0.544
weib	0.026	0.065	0.27	0.544	

Table A5. Dot-motion direction discrimination recovery bias (c) for all models . Numbers refer to the bias for the row-model and against the column-model (with c being sign-reversed across the diagonal).

	exp3	*exp4*	*pow3*	*pow4*	*weib*
exp3		0.529	2.386	4.406	0.226
exp4	-0.103		1.706	3.883	-0.256
pow3	0.147	-0.121		2.161	-0.2
pow4	-1.91	-1.86	-1.737		-2.065
weib	0.259	0.441	2.07	4.592	

Table A6. Dot-motion direction discrimination recovery T values for all models
